# Organization of head and neck cancer rehabilitation care: a national survey among healthcare professionals in Dutch head and neck cancer centers

**DOI:** 10.1007/s00405-024-08488-1

**Published:** 2024-02-07

**Authors:** Ellen Passchier, Ann-Jean C. C. Beck, Martijn M. Stuiver, Valesca P. Retèl, Arash Navran, Wim H. van Harten, Michiel W. M. van den Brekel, Lisette van der Molen, the Dutch Head, Neck Society

**Affiliations:** 1 Department of Head and Neck Oncology and Surgery, The Netherlands Cancer Institute, Plesmanlaan 121, 1066 CX Amsterdam, The Netherlands; 2 Division of Psychosocial Research and Epidemiology, The Netherlands Cancer Institute, Amsterdam, The Netherlands; 3 Department of Health Technology and Services Research, University of Twente, Enschede, The Netherlands; 4 Department of Radiation Oncology, Netherlands Cancer Institute, Amsterdam, The Netherlands; 5 Institute of Phonetic Sciences ACLC, University of Amsterdam, Amsterdam, The Netherlands; 6 Department of Oral and Maxillofacial Surgery, Amsterdam UMC, Amsterdam, The Netherlands; 7 Centre for Quality of Life, The Netherlands Cancer Institute, Amsterdam, The Netherlands; 8 Center of Expertise Urban Vitality, Faculty of Health, Amsterdam University of Applied Sciences, Amsterdam, The Netherlands

**Keywords:** Survey, Rehabilitation, Head and neck cancer, Survivorship care, Barriers and facilitators, The Netherlands

## Abstract

**Purpose:**

Head and neck cancer (HNC) treatment often leads to physical and psychosocial impairments. Rehabilitation can overcome these limitations and improve quality of life. The aim of this study is to obtain an overview of rehabilitation care for HNC, and to investigate factors influencing rehabilitation provision, in Dutch HNC centers, and to some extent compare it to other countries.

**Methods:**

An online survey, covering five themes: organizational structure; rehabilitation interventions; financing; barriers and facilitators; satisfaction and future improvements, among HNC healthcare- and financial professionals of Dutch HNC centers.

**Results:**

Most centers (86%) applied some type of rehabilitation care, with variations in organizational structure. A speech language therapist, physiotherapist and dietitian were available in all centers, but other rehabilitation healthcare professionals in less than 60%. Facilitators for providing rehabilitation services included availability of a contact person, and positive attitude, motivation, and expertise of healthcare professionals. Barriers were lack of reimbursement, and patient related barriers including comorbidity, travel (time), low health literacy, limited financial capacity, and poor motivation.

**Conclusion:**

Although all HNC centers included offer rehabilitation services, there is substantial practice variation, both nationally and internationally. Factors influencing rehabilitation are related to the motivation and expertise of the treatment team, but also to reimbursement aspects and patient related factors. More research is needed to investigate the extent to which practice variation impacts individual patient outcomes and how to integrate HNC rehabilitation into routine clinical pathways.

**Supplementary Information:**

The online version contains supplementary material available at 10.1007/s00405-024-08488-1.

## Introduction

In the Netherlands, approximately 3100 patients are diagnosed with head and neck cancer (HNC) annually [1]. HNC treatment can result in diverse and high morbidity, such as eating and swallowing problems, altered speech, xerostomia, shoulder disability, fatigue, severe deconditioning and/or psychosocial problems. Although high quality evidence is still scarce on what constitutes an optimal rehabilitation program for people with HNC in terms of modalities, organization and timing, several studies have reported positive outcomes of rehabilitation programs. This includes improved function, better coping with potential handicaps and optimized functional outcomes and quality of life [2–6].

HNC treatment is often complex and requires a multidisciplinary approach. Therefore, in the Netherlands, HNC care is centralized in eight academic/cancer centers and six satellite centers recognized by the Dutch Head and Neck Society (DHNS). The DHNS strives to improve quality and harmonize care of HNC across all centers, based on the best available evidence. While medical treatment in these centers is uniform to a high degree due to national guidelines, allied health care and rehabilitation treatment is not, leaving room for practice variation.

Practice variation can have impact on the quality of care, resulting in variation in functional outcomes [7]. In the past decade, there have been various initiatives to harmonize and improve rehabilitation care in HNC patients, focusing on needs assessment, rehabilitation interventions, functional outcome assessment, and quality of life [3, 6, 8–11]. The DHNS guideline further recommends providing guidance in smoking and alcohol cessation; screening for and provide psychosocial guidance during the cancer care continuum and referral to a psychologist or psychiatrist if necessary. However, the guideline does not give any recommendations with regard to triage, which evidence-based interventions to use, or how to evaluate rehabilitation outcomes [12]. Though, there is a Dutch general cancer rehabilitation guideline available, which provides recommendations for screening, triage and interventions for people with cancer, regardless of tumor type [13].

To date, it remains unclear to what extent these guidelines are applied in routine practice in the Netherlands. Moreover, within the Dutch healthcare system, there is variation in coverage of rehabilitation cancer care, due to differences in the extent to which patients themselves are insured and due to substantive choices in financing care pathways [14]. It is, therefore, also of interest to investigate factors associated with provision and financing of rehabilitation care.

Obtaining insight into the current organization and content of rehabilitation care provided in HNC centers is important to map practice variation, to identify barriers and facilitators for provision of rehabilitation services, and to define future aspirations for HNC rehabilitation development and implementation. Currently, an overview including these aspects is lacking. Therefore, the main purpose of this survey study is to give an overview of the organizational structure and content of rehabilitation care, and financial matters, in the context of HNC rehabilitation as offered in the Dutch HNC centers. In addition, we explore factors influencing HNC rehabilitation provision and identify opportunities for optimization of HNC rehabilitation care.

## Methods

### Study design and participating centers

In collaboration with the DHNS we disseminated a survey, by sending a link, which referred to an online platform. All 14 Dutch HNC centers were approached through the DHNS. Professionals involved in the clinical or financial processes of HNC rehabilitation completed the online survey in 2018.

### Ethical considerations

The study was submitted to the hospital institutional review board, no ethical approval was needed, and no patients were included.

### Framework of the survey

In this cross-sectional survey study, the term cancer rehabilitation was used as defined by the (Dutch) National Health Care Institute (ZIN): ‘care that focuses on functional, physical, psychological and social problems related to cancer, including supportive and rehabilitation care’ [15]. Accordingly, ‘rehabilitation’ is used as an umbrella term for all mono-, multi-, and interdisciplinary care with that focus.

We constructed a self-administrated survey, consisting of 121 multiple choice and open questions. The first questions (*n =* 5) queried information on characteristics of survey respondents. Respondents were referred to discipline specific sections of the questionnaire based on their profession.

The survey was developed in collaboration with a panel of experts consisting of medical specialists and healthcare professionals involved in rehabilitation including the Dutch working group of head and neck cancer allied healthcare professionals (*Paramedische Werkgroep Hoofd-halstumoren*—PWHHT) (Sects. 1, 2, 4, and 5), managers/employees of the Financial Department and business specialists of a healthcare insurer (Sect. 3 and 4).

The Chairman of each DHNS per center indicated which of the allied health professionals were involved in rehabilitation care in the institute and forwarded the survey link to these disciplines. One representative of each available allied health care provider filled in the discipline specific part of the survey. The online platform required completion of all items, which resulted in no missing values. Items were either multiple choice, scored on a four-point scale (always/often/sometimes/never) or on a dichotomous scale (yes/no), open questions were added for clarification. If sections were completed in duplicate for a center (i.e., because the survey link was sent to and responded by multiple representatives) and showed discrepancies, these were resolved by email contact with both responders. An English translation of the survey is available in Appendix A.

#### Section 1: organizational structure

The first part of Sect. 1 of the survey was set up to obtain information on rehabilitation provision, protocols or guidelines used, timing of rehabilitation and tumor or treatment related factors (six items). The second and third part of Sect. 1 were designed to assess to what extent the Dutch cancer rehabilitation guidelines [16] were applied in the context of HNC care. This section included questions on the rehabilitation processes, screening, intake and referral (twelve items), as well as evaluation (ten items). The questions in this section were completed by a medical specialist or oncology nurse specialist as a representative of the HNC multidisciplinary team of each center.

#### Section 2: content of rehabilitation care

Section 2 included content of rehabilitation services and was completed by the representative of each allied health care profession present in the center. We assessed the availability of allied health care professionals in each center, as well as any applied rehabilitation modules. For all allied health care professions, we collected information on which interventions and clinimetric tools for HNC rehabilitation were available and used. The inventory included the involvement of the following allied health care professionals: a physiotherapist, speech-language therapist, dietitian, occupational therapist, medical social worker, psychologist, psychiatrist/psychiatric nurse (specialist) and an art therapist. These disciplines were chosen predicated upon DHNS guideline recommendations, and a hospital based multidisciplinary head and neck rehabilitation program [3].

#### Section 3: financial matters

In Sect. 3, reimbursement was addressed, using multiple-choice questions. For each hospital, we inventoried whether reimbursement was available and sufficient or if there were any financial restrictions to provide rehabilitation. This part was completed by financial controllers or managers.

#### Section 4: factors influencing rehabilitation provision

Barriers to and facilitators of rehabilitation provision in Sect. 4 were categorized into clinician related, economic, patient-related and organizational categories [17]. Each category was covered by multiple items (43 items) reflecting different aspects. Respondents were asked to identify each aspect in the past six months as being either a ‘barrier’ or ‘facilitator’. Other options were ‘I don’t know’ or ‘does not occur regularly in my situation’. This section was completed by all clinicians. Additionally, medical specialists responded to two items within the economic domain regarding coverage and cost evaluation, and financial controllers or managers responded to the economic category concerning budget negotiation with health insurers (three items). An overview of all 43 items is provided in Appendix B.

#### Section 5: satisfaction with rehabilitation and suggestions for improvement

**Fig. 1:**
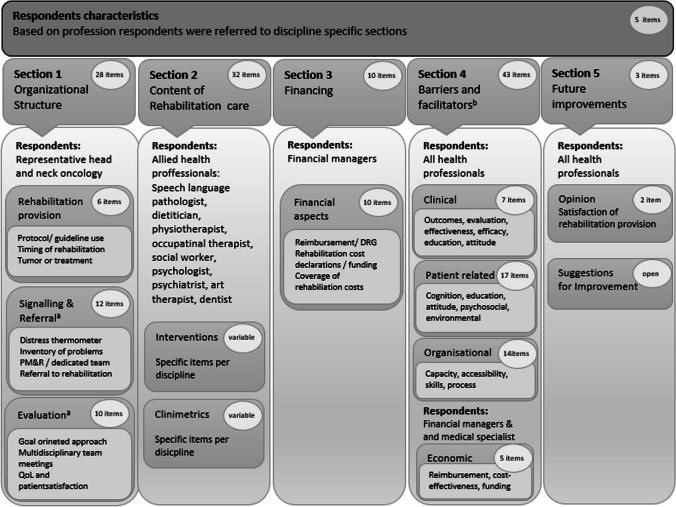
Structure and content of the survey. Survey respondents: The representative of the team was a chairman of the DHNS (head and neck surgeon, radiotherapist), nurse specialist or PM&R physician. If available, a representative of the rehabilitation team answered items related to the discipline. The representative of the Financial Department comprised of an manager or employee of the department. ^a^Based on recommendations guideline cancer rehabilitation. ^b^Barriers and facilitators were based on domains of Institute of Medicine (IOM). PM&R, physical medicine and rehabilitation; QoL, quality of life; DHNS, Dutch Head and Neck Society

We added two items on satisfaction, and an open questions to provide suggestions for future improvements (Sect. 5). Details on the design and background of the survey are visualized in Fig. 1.

### Data analysis

Descriptive statistics were generated to summarize respondent characteristics and the responses for all sections. Barriers and facilitators were quantitatively ranked by most commonly endorsed items and visualized in radar plots. The open questions from Sect. 5 were discussed in the research team and categorized based on consensus. Analysis and plots were conducted in IBM SPSS Statistics 22 and Excel (*Microsoft Corporation. (2018). Microsoft Excel)*.

## Results

### Characteristics of survey respondents

All HNC centers—eight main HNC academic cancer centers and six affiliated non-academic centers – were approached and responded in full. We had 113 respondents, most were female (73%, 82/113), and their mean work experience was 12.0 (1–40) years.

Section 1 was completed by head and neck surgeons mostly (48%, 13/27 respondents). Seventy healthcare professionals completed Sect. 2, of which 23% were speech language therapists (16/70), 21% dietitians (15/70), 19% physiotherapists (13/70), 13% medical social workers (9/70), 10% psychologists (7/70) and 14% others (10/70). Section 3 was completed by DBC (*diagnose behandel combinatie* – Dutch diagnosis-related group (DRG)) consultants (44%, 7/16 respondents); employees of the financial department (31%, 5/16); and managers (25%, 4/16). An overview of all respondents’ characteristics is provided in Table 1.

**Table 1:** Characteristics of survey respondents of 14 Dutch head and neck cancer centers

	Respondents to Sect. 1: Organizational structure (%)	Respondents to Sect. 2: Rehabilitation modules (%)	Respondents to Sect. 3: Financial matters (%)	Total respondents (%)
Number of respondents (n)	27	70	16	113
Mean age, y (range)	50.4 (31–64)	45.1 (24–64)	40.3 (25–63)	45.7 (24–64)
Sex				
Male	13 (48.1)	12 (17.1)	5 (31.3)	30 (26.5)
Female	14 (51.9)	58 (82.9)	11 (68.8)	83 (73.5)
Function				
Head and neck surgeon	13 (48.1)			13 (11.5)
Nurse specialist	12 (44.4)			12 (10.6)
Radiotherapist	1 (3.7)			1 (0.9)
PM and R physician	1 (3.7)			1 (0.9)
Art therapist		2 (2.9)		2 (1.8)
Dietitian		15 (21.4)		15 (13.3)
Medical social worker		9 (12.9)		9 (8.0)
Occupational therapist		3 (4.3)		3 (2.7)
Psychiatrist		1 (1.4)		1 (0.9)
Psychiatric nurse (specialist)		4 (5.7)		4 (3.5)
Psychologist		7 (10.0)		7 (6.2)
Physiotherapist		12 (17.1)		13 (11.5)
Speech-language therapist		16 (22.9)		16 (14.2)
DBC* consultant			7 (43.8)	7 (6.2)
Employee financial Department			5 (31.3)	5 (4.4)
Manager financial department			4 (25)	4 (3.5)
Institute				
Academic/cancer center	13 (48.1)	46 (65.7)	10 (62.5)	69 (61.1)
Non-academic center	14 (51.9)	24 (34.3)	6 (37.5)	44 (38.9)
Mean work experience, y (range)	14.3 (2–32)	12.4 (1–40)	6.3 (1–20)	12.0 (1–40)

DBC, *diagnose behandel combinatie*;

PM&R physician, physical medicine and rehabilitation physician

^*^A DBC is a Dutch diagnosis-related group (DRG)

### Survey results

#### Section 1: Organizational structure

Rehabilitation (mono-, multi, or interdisciplinary) care was provided on a routine basis in all centers, either according to a protocol (64%, 9/14 centers), indicated by needs assessment (21%, 3/14) or both (14%, 2/14). Rehabilitation was initiated in all treatment phases: before treatment (93%, 13/14), during treatment (57%, 8/14) and/or after treatment (57%, 8/14). The extent of rehabilitation provision was dependent on tumor type (most often advanced stage laryngeal, oropharyngeal and nasopharyngeal cancer) in four centers (29%, 4/14), and on treatment modality (radiotherapy or surgery and chemo radiation) in two centers (14%, 2/14). All centers (except one) systematically screened for physical and/or psychosocial needs. Most centers (86%, 12/14) used the distress thermometer [18] combined with a consultation for this purpose, and 4 centers used Quality of Life questionnaires in addition. In all centers, screening for distress and/or referral was carried out by a nurse (practitioner) or case manager, in eight centers additionally by a medical specialist and in six centers by an allied health professional. Various national and/or local guidelines and protocols were used for rehabilitation needs assessment and referral, of which the national guideline on detecting the need for psychosocial care (57%, 8/14) and department’s local protocols (43%, 6/14) were most applied (Appendix C).

#### Section 2: Rehabilitation interventions

A detailed overview of clinimetrics used and interventions available per discipline for each center is provided in Appendix D. A speech-language therapist was available in all centers. Most centers also provided HNC rehabilitation care by a dietitian (93%, 13/14), and physiotherapist (86%, 12/14). A medical social worker and a psychologist were involved in HNC rehabilitation in eight and seven centers, respectively. Other professionals were involved in rehabilitation less frequently: a psychiatrist or psychiatric nurse (specialist) was involved in five centers (36%), an occupational therapist in three (21%), and an art therapist in two centers (14%). Most centers (79%, 11/14) reported to refer patients either within the hospital or to external health care professionals when these were not available for delivering HNC rehabilitation services. Health care providers not routinely involved in HNC rehabilitation care included physiotherapy (2 centers), nutritional care (1 center), occupational therapy (4 centers), psychology (6 centers), and psychiatry/psychiatric nursing (7 centers). Interventions and clinimetrics applied by represented allied health professionals varied extensively. For example, *swallowing rehabilitation* by a speech language therapist was provided as standard care in 71% (10/14), while in 29% (4/10), patients were referred only after an indication by needs assessment. A swallowing video or FEES was performed as standard measurement for *swallowing rehabilitation* in only two centers (14%) and in the remaining 12 centers (86%) if indicated after consultation by the speech language therapist.

#### Section 3: Financial matters

Only one center covered most rehabilitation costs with a rehabilitation-specific Dutch diagnosis-related group (DRG). In three centers, costs were sufficiently covered by other various sources. In five other centers this was not the case, and in six centers this was unknown. For three non-university centers, the lack of reimbursement was the reason for referring patients with rehabilitation needs to primary care. In two centers, reimbursement of rehabilitation care was insufficient because of the unavailability of a DRG and use of maximum tariffs. In one center, dental care and provision of rehabilitation during the follow-up period in the outpatient clinic by healthcare professionals (e.g., speech language therapist, physiotherapist) were not refunded.

#### Section 4: Barriers and facilitators

All invited healthcare professionals in 14 HNC centers, 97 respondents in total, completed Sect. 4 on barriers and facilitators. In addition, 15 respondents of the Financial Department of all 14 HNC centers completed the second part of the economic items.

The 97 health care professionals scored all items more often as being a facilitator (average number of respondents per item *n =* 42 of 97, 44%) rather than a barrier (*n =* 18 of 97, 19%). Items were most frequently reported as facilitators within the clinician-related category, while most perceived barriers were patient-related (Table 2).

**Table 2:** Top 7 barriers and facilitators of providing head and neck cancer rehabilitation care completed by 97 survey respondents in 14 Dutch centers

No	Category	Barrier (no. of respondents)	No.	Category	Facilitator (no. of respondents)
1	Patient-related	Psychiatric history and/or comorbidity (59)	1	Clinician-related	Attitude of HP towards rehabilitation (76)
2	Patient-related	Travel time to/from the hospital (53)	2	Clinician-related	Motivation of HP to provide rehabilitation (76)
3	Patient-related	Transport to/from the hospital (52)	3	Clinician-related	Expertise/knowledge of specialists and HP (73)
4	Patient-related	Health literacy of patients (45)	4	Organizational	Availability of a contact person for patients (69)
5	Patient-related	Financial capacity of patients (43)	5	Clinician related	Attitude of specialists towards rehabilitation (67)
6	Economic	Reimbursement structure for rehabilitation (36)	6	Clinician related	Knowledge of HP (in general) on rehabilitation (67)
7	Patient-related	Motivation and therapy compliance of patients (35)	7	Patient-related	Availability patient information on rehabilitation (67)

**Fig. 2:**
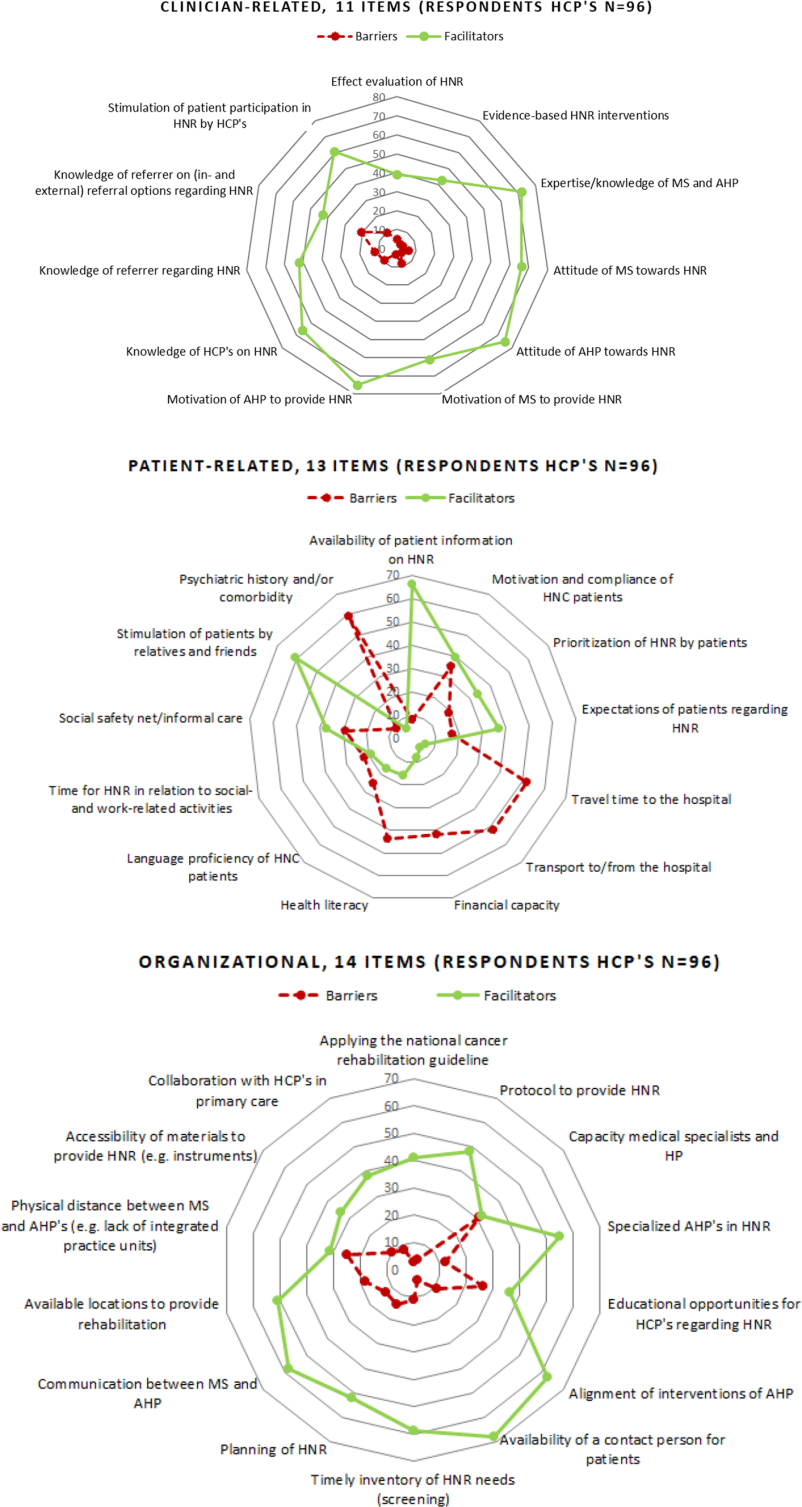
Barriers and facilitators plotted per category Clinician-related, Patient-related, and Organizational and plotted per item. Each plot displays (axis %) the barriers or facilitators reported by the respondents

Clinician-related factors scored by the respondents as facilitators included a positive attitude towards HNC rehabilitation and the available expertise/knowledge of healthcare professionals in the HNC team. Endorsed organizational facilitators included informing patients about HNC rehabilitation possibilities and the presence of a contact person. Patient-related factors that were scored as barriers were related to patients’ medical history (e.g., psychiatric disease), travel (-time), low health literacy and poor motivation. Lack of reimbursement for rehabilitation was also reported as a barrier. Figure 2 shows how health care professionals interpreted items as barriers or facilitators for the categories clinician-related, patient-related and organizational.

Financial support from the hospital was mentioned as a facilitating factor in one hospital. In contrast, three other centers reported barriers on negotiated tariffs, hospital’s financial support/grants and contractual agreements.

#### Section 5: Health professionals’ satisfaction and suggestions for future improvements

Eighty-nine percent of all health care professionals (*n =* 97) agreed that a multi-/interdisciplinary rehabilitation program, might be of added value compared to less integrated rehabilitation services. Nevertheless, most health care professionals (70%) were (very) satisfied with their current rehabilitation provision. Six respondents (5%) were (very) dissatisfied and 25% reported neutral opinion.

Suggestions for improvements were related to: (1) improving (availability of) multi-/interdisciplinary rehabilitation (*n =* 23), i.e., including multidisciplinary team meetings; improving alignment and coordination; (2) patient screening (*n =* 18), i.e., a systematic needs assessment including use of Patient Reported Outcome Measurements (PROMs) in combination with consultation by a specialized HNC nurse or nurse specialist as a case manager; (3) standard and/ or early consultation by healthcare professionals (*n =* 9), i.e., with a speech language therapist, physiotherapist, psychiatrist and/or psychosocial care provider; (4) consultation of healthcare professionals during the follow-up period and cancer care continuum (*n =* 6); (5) reimbursement of rehabilitation (*n =* 8), e.g., by means of improving the DRG structure and coverage of supportive care in primary health care—as well as funding of care for relatives.

## Discussion

This survey study, with a response of 113 medical, allied health, and financial professionals from all Dutch HNC centers, provides a comprehensive overview of organization, content and financing of HNC rehabilitation in the Netherlands. All Dutch HNC centers reported to provide some form of HNC rehabilitation care, though results indicate considerable variation.

Several protocols were applied, varying from hospital-based protocols to the general national guideline for cancer rehabilitation [13]. The latter guideline suggests that multidisciplinary rehabilitation should be offered to patients who experience interrelated health problems in multiple domains (i.e., physical and psychosocial), which is often the case after HNC treatment. The efficacy of multidisciplinary HNC rehabilitation care has not yet been established; however, there is sufficient evidence that HNC rehabilitation interventions can have a positive effect on functional outcomes, coping and quality of life [4, 10, 11, 19].

Guidelines and literature recommend a speech language therapist (SLT) to be standard part of HNC care, which is indeed the case in all Dutch HNC centers[16, 20]. However, interventions and clinimetrics used by SLTs vary substantially across centers. For example, although the effectiveness of (preventive) swallowing rehabilitation in HNC patients has been demonstrated [21–23], this survey showed that swallowing rehabilitation is not yet standard care in all HNC centers, despite the availability of SLTs. Our results showed even larger differences in deployment of other allied health care professionals and the clinimetrics and rehabilitation interventions they use. Underutilization of rehabilitation services by SLT’s, physical therapists (PT) and occupational therapists (OT), were also found in a study performed in the United States [24]. Consequently, there is a risk that the observed variation in availability and use of (evidence-based) interventions lead to insufficient rehabilitation care, and thereby to poorer outcomes, for some patients [24].

Except for one center, all centers routinely screened for physical or psychosocial problems, although timing of needs assessment, screening tool or PROMs used, and additional follow-up consultations varied. The importance of screening for psychosocial problems is generally accepted because of the high prevalence of e.g. depression and anxiety [25], and problems in the social domain like financial toxicity [26, 27] and problems with return to work [28] in people with HNC. However, at the same time, high level evidence on how to solve these problems is still scarce [29, 30].

The respondents endorsed that organization of HNC rehabilitation care should ideally be carried out in a multidisciplinary team setting, as recommended in a recent publication by the European Head and Neck Society (EHNS) and previously by the American Head and Neck Society (AHNS) [10, 31]. An integrated multidisciplinary team should address specific HNC rehabilitation needs, and emphasize the need for psychosocial support [20]. However, this survey shows psychosocial rehabilitation care was underrepresented in Dutch HNC centers. Furthermore, if health problems are interrelated and/or severe, which often occurs in HNC patients, multidisciplinary or integrated rehabilitation coordinated by physiatrist is also recommended by the Dutch national guideline on cancer rehabilitation [13]. At the same time, such a setting was available in only two centers in this study. Although there is no conclusive evidence of the value of an integrated approach, given the complexity of HNC and the requirement of qualified HNC rehabilitation care professionals, it is questionable whether optimal HNC rehabilitation can be provided in primary health care. More likely, it should be centralized like oncological treatment of head and neck cancer to ensure that the involved health care professionals can build and maintain sufficient clinical expertise [20, 31–33].

Substantial variation was also present for financial coverage of HNC rehabilitation services. Only one center had funding through a rehabilitation-specific DRG; others used various, usually less secure, funding sources. This national variation in reimbursement might limit accessibility to rehabilitation care, and consequently to inequity [17].

Both the AHNS and EHNS recommend an integrated clinical care team to manage the ongoing care needs of HNC survivors and to develop a personalized rehabilitation or survivorship plan, considering functional as well as psychosocial consequences [20, 31]. To realize this ideal approach, several challenges need to be overcome. The factors that were reported in this survey study might help pinpoint which facilitators could be leveraged and which barriers need to be addressed to optimize organization of rehabilitation HNC services.

We observed that in general, the positive attitude and expertise of healthcare professionals was perceived as facilitating. Most perceived barriers reported in our study were patient-related. On the one hand, this is not surprising, as HNC patients are often vulnerable, have many comorbidities and limited social support, low financial resources and poor health literacy [27]. On the other hand, from the perspective of patient-centered care and health-equity, such factors should be explicitly addressed in the design of rehabilitation services, rather than being considered unamenable barriers. While on a macro-level, standardization across centers is desirable, on the micro-level, a personalized assessment of rehabilitation needs and subsequent supportive interventions that address not only health care needs but also specific barriers to pursue and achieve an improved health condition are necessary [5, 10, 19, 34]. A recent scoping review identified comparable barriers and facilitators that influence access, referral and rehabilitation participation for patients with head and neck or lung cancer. Access was influenced by variation in needs. HCPs positive attitude, knowledge and availability of HCPs enhanced patients access. Lack of knowledge and lack of integrated teams were found as barriers to rehabilitation access. Patient-centered care, and rehabilitation care embedded within the cancer care pathway, guideline implementation, and reimbursement were identified as facilitating rehabilitation access. Referral to rehabilitation was negatively influenced due to unclear eligibility criteria, and inconsistent referral pathways. Main barriers for participation in rehabilitation services were timing, burden of impairment, and socioeconomic status [33]. The functional impairments, acute toxicity, and late effects resulting from HNC treatment, in a vulnerable and heterogenetic population, make regular needs and health-related quality of life assessment, with timely referral throughout the cancer care continuum, indispensable. To support early identification of risks and timely management of functional, physical, emotional and socioeconomic factors, PROMS are recommended to be incorporated in care pathways [17, 35–37]. However, PROMS would not help those with low literacy and are therefore not sufficient to achieve equitable access to care. A case manager can have a pivotal role in managing unmet needs and personal barriers, support self-management, and stimulate healthy behavior [5, 20, 38]. In addition, the quality of information provision and monitoring of rehabilitation needs could be enhanced by using multimedia resources, and e-health platforms, carefully designed to also reach those with poor (health) literacy [39]. To address pre-existing risk-factors resulting from comorbid conditions, prehabilitation may be a way to optimize management of both cancer and functional outcomes, although evidence for this approach is still limited, especially for HNC care [40].

Economic barriers were reported as the second most important factor, and reimbursement of HNC rehabilitation is mostly not fully available. Referral to care givers close to the house address reduces travel costs, but such care is often insufficiently covered by insurance and might increase financial toxicity [26, 41, 42].

The main strengths of the present study were the full response from stakeholders of all Dutch HNC centers, and the absence of missing data. First the results presented in this paper only reflect the current situation at the time of the survey, in 2018. During a meeting of the DHNS rehabilitation-working group, the results were checked and we concluded that it is unlikely that major changes have occurred. By its nature, a survey study also has limitations. The answers are prone to subjectivity and answers are limited to the answering options provided. The inventory was set up from a general interdisciplinary cancer rehabilitation perspective, and the health-care professionals queried were chosen accordingly. Consequently, we did not include dental care (e.g., maxillofacial prosthodontist) and other professionals (e.g., lymphedema therapists) in our survey sample, even though these professionals could also provide rehabilitation care in the context of HNC [16, 43]. Additionally, this survey was conducted only from the health care provider perspective. Future qualitative research such as individual semi-structured interviews or focus group discussions with HNC patients, their caregivers and professionals could be useful to obtain more in-depth information.

## Conclusion

All 14 Dutch Head and Neck Centers provide some form of rehabilitation (e.g. mono, multi- or interdisciplinary), and most apply a protocol to identify, triage and refer patients to rehabilitation interventions. Nevertheless, there is substantial practice variation in HNC rehabilitation in the Netherlands, with respect to needs assessment, protocol use, referral to and availability and timing of rehabilitation care, as well as financing such care. Recent guidelines by the AHNS and EHNS warrant further efforts to improve equitable access to rehabilitation services for people with HNC in the Netherlands and abroad.

### Supplementary Information

Below is the link to the electronic supplementary material.

Supplementary file1 (PDF 354 KB)

Supplementary file2 (PDF 418 KB)

Supplementary file3 (PDF 211 KB)

Supplementary file4 (PDF 653 KB)

## Data Availability

The data will be available upon request (contact person: E. Passchier: e.passchier@nki.nl).

## Abbreviations

HCP’s: Health care professionals
HNR: Head and neck rehabilitation
MS: Medical specialist
AHP: Allied health professionals

## References

[1] IKNL kankerregistratie. 2023. https://iknl.nl/kankersoorten/hoofd-halskanker/registratie/incidentie.

[2] Parke SC, Langelier DM, Cheng JT, Kline-Quiroz C, Stubblefield MD (2022). State of rehabilitation research in the head and neck cancer population: functional impact vs impairment-focused outcomes. Curr Oncol Rep.

[3] Passchier E, Stuiver MM, van der Molen L, Kerkhof SIC, van den Brekel MWM, Hilgers FJM (2016). Feasibility and impact of a dedicated multidisciplinary rehabilitation program on health-related quality of life in advanced head and neck cancer patients. Eur Arch Otorhinolaryngol.

[4] Eades M, Murphy J, Carney S, Amdouni S, Lemoignan J, Jelowicki M, Nadler M, Chasen M, Gagnon B (2013). Effect of an interdisciplinary rehabilitation program on quality of life in patients with head and neck cancer: review of clinical experience. Head Neck.

[5] Ringash J, Bernstein LJ, Devins G, Dunphy C, Giuliani M, Martino R, McEwen S (2018). Head and neck cancer survivorship: learning the needs, meeting the needs. Semin Radiat Oncol.

[6] Ward EC, Van As-Brooks CJ (2014). Head and neck cancer: treatment, rehabilitation, and outcomes.

[7] Wouters MW, Jansen-Landheer ML, van de Velde CJ (2010). The quality of cancer care initiative in the Netherlands. Eur J Surg Oncol (EJSO)..

[8] Verdonck-de Leeuw IM (2009). Computerized prospective screening for high levels of emotional distress in head and neck cancer patients and referral rate to psychosocial care. Oral Oncol.

[9] Verdonck-de Leeuw I (2019). Advancing interdisciplinary research in head and neck cancer through a multicenter longitudinal prospective cohort study: the NETherlands QUality of life and BIomedical Cohort (NET-QUBIC) data warehouse and biobank. BMC Cancer.

[10] Goyal N (2022). Head and neck cancer survivorship consensus statement from the American Head and Neck society. Laryngosc Investig Otolaryngol.

[11] Rodriguez AM, Komar A, Ringash J, Chan C, Davis AM, Jones J, Martino R, McEwen S (2019). A scoping review of rehabilitation interventions for survivors of head and neck cancer. Disabil Rehabil.

[12] Federatie van medisch specialisten. Guideline head and neck tumors (Dutch). 2014. https://richtlijnendatabase.nl/richtlijn/hoofd-halstumoren_sept_2023/follow-up_nazorg/follow-up_behandeling_hoofd-halstumoren.html

[13] Federatie van medisch specialisten. National guideline on cancer rehabilitation (Dutch). 2017. https://richtlijnendatabase.nl/richtlijn/medisch_specialistische_revalidatie_bij_oncologie/algemeen.html.

[14] Pearce A, Tomalin B, Kaambwa B, Horevoorts N, Duijts S, Mols F, van de Poll-Franse L, Koczwara B (2019). Financial toxicity is more than costs of care: the relationship between employment and financial toxicity in long-term cancer survivors. J Cancer Surviv.

[15] Zorginstituut Nederland. Standpunt-medisch-specialistische-revalidatie---zorg-zoals-revalidatieartsen-plegen-te-bieden. 2015. https://www.zorginstituutnederland.nl/publicaties/standpunten/2015/06/22/standpunt-medisch-specialistische-revalidatie---zorg-zoals-revalidatieartsen-plegen-te-bieden.

[16] Dutch head and neck society. Hoofd_halstumoren_richtlijnenddatabase_betreft ondersteunende zorg aanbevelingen.pdf. 2022.

[17] Wolfe A (2001). Institute of Medicine (US) Committee on Quality of Health Care in America., Institute of Medicine Report: crossing the quality chasm: a new health care system for the 21st century, in Policy. Politics Nurs Pract.

[18] Dutch version of the distress thermometer; de Lastmeter. 2008. https://www.kanker.nl/hulp-en-ondersteuning/lastmeter.

[19] Barnhart MK, Ward EC, Cartmill B, Nund R, Robinson RA, Chandler SJ, Smee RI (2019). Content analysis of rehabilitation goals for patients following non-surgical head and neck cancer treatment. Support Care Cancer.

[20] Verdonck-de Leeuw I, Dawson C (2022). European Head and Neck Society recommendations for head and neck cancer survivorship care. Oral Oncol.

[21] Clarke P, Radford K, Coffey M, Stewart M (2016). Speech and swallow rehabilitation in head and neck cancer: United Kingdom National Multidisciplinary Guidelines. J Laryngol Otol.

[22] Van der Molen L, van Rossum MA, Burkhead LM, Smeele LW, Hilgers FJM (2009). Functional outcomes and rehabilitation strategies in patients treated with chemoradiotherapy for advanced head and neck cancer: a systematic review. Eur Arch Otorhinolaryngol.

[23] Brady R, McSharry L, Lawson S, Regan J (2022). The impact of dysphagia prehabilitation on swallowing outcomes post-chemoradiation therapy in head and neck cancer: a systematic review. Eur J Cancer Care.

[24] Wang JR (2019). Utilization of rehabilitation services in patients with head and neck cancer in the United States: a SEER-Medicare analysis. Head Neck.

[25] Hammermüller C, Hinz A, Dietz A (2021). Depression, anxiety, fatigue, and quality of life in a large sample of patients suffering from head and neck cancer in comparison with the general population. BMC Cancer.

[26] Hueniken K, Douglas CM, Jethwa AR (2020). Measuring financial toxicity incurred after treatment of head and neck cancer: development and validation of the financial index of toxicity questionnaire. Cancer.

[27] Rogers SN, Lowe D, Kanatas A (2021). Social determinants of health-related quality of life outcomes for head and neck cancer patients. Oral.

[28] Zecena Morales C, Karolina L, McDowell L, Piper A, Jefford M (2023). Return to work in head and neck cancer survivors: a systematic review. J Cancer Surviv.

[29] Giuliani M, Papadakos J, Broadhurst M (2019). The prevalence and determinants of return to work in head and neck cancer survivors. Support Care Cancer.

[30] Richardson AE, Broadbent E, Morton RP (2019). A systematic review of psychological interventions for patients with head and neck cancer. Support Care Cancer.

[31] Cohen EE (2016). American Cancer Society head and neck cancer survivorship care guideline. CA Cancer J Clin.

[32] Kilsdonk MJ, Siesling S, van Dijk BA, Wouters MW, van Harten WH (2018). What drives centralisation in cancer care?. PLoS ONE.

[33] Navntoft S (2023). Barriers and facilitators to cancer rehabilitation for patients with head and neck or lung cancer—a scoping review mapping structural and healthcare professionals’ perspectives. Disabil Rehabil.

[34] Kanatas A, Lowe D, Rogers SN (2022). Health-related quality of life at 3 months following head and neck cancer treatment is a key predictor of longer-term outcome and of benefit from using the patient concerns inventory. Cancer Med.

[35] Shunmugasundaram C, Rutherford C, Butow PN, Sundaresan P, Dhillon HM (2019). Content comparison of unmet needs self-report measures used in patients with head and neck cancer: a systematic review. Psychooncology.

[36] de Jel DV, Young-Afat DA, Ooms-Renckens MM, Smeele LE, Rakhorst HA (2023). Patients' and healthcare professionals' perspectives on better use of patient-reported outcome measures in head and neck cancer. Value Health.

[37] De Felice F, Tombolini V, de Vincentiis M (2019). Multidisciplinary team in head and neck cancer: a management model. Med Oncol.

[38] Dempsey L, Orr S, Lane S, Scott A (2016). The clinical nurse specialist's role in head and neck cancer care: United Kingdom national multidisciplinary guidelines. J Laryngol Otol.

[39] van Overveld LF, Takes RP, Vijn TW (2017). Feedback preferences of patients, professionals and health insurers in integrated head and neck cancer care. Health Expect.

[40] Faithfull S, Turner L, Poole K (2019). Prehabilitation for adults diagnosed with cancer: a systematic review of long-term physical function, nutrition and patient-reported outcomes. Eur J Cancer Care.

[41] Mady LJ (2019). Understanding financial toxicity in head and neck cancer survivors. Oral Oncol.

[42] Khan MN, Hueniken K, Manojlovic-Kolarski M (2022). Out-of-pocket costs associated with head and neck cancer treatment. Cancer Rep..

[43] Vosselman N, Alberga J, Witjes MHJ (2021). Prosthodontic rehabilitation of head and neck cancer patients—challenges and new developments. Oral Dis.

