# Supplementary material for: Organization of head and neck cancer rehabilitation care: a national survey among healthcare professionals in Dutch head and neck cancer centers
Source: Eur Arch Otorhinolaryngol. 2024 Feb 7;281(5):2575–85. doi: 10.1007/s00405-024-08488-1 (PMC11023954; doi:10.1007/s00405-024-08488-1)
Supplement: Supplementary file 1 — Supplementary file1 (PDF 354 KB) [file 405_2024_8488_MOESM1_ESM.pdf]

SURVEY: REHABILITATION IN HEAD AND NECK CANCER

Dear Colleague,

This survey was developed to gain insight into the way in which rehabilitation for head and neck cancer patients (HNSCC), who are treated with curative intention, is organized in the head and neck centers in the Netherlands.

In this survey we defined rehabilitation care as:

'care that focusses on functional, physical, psychological and social problems related to cancer, including supportive and rehabilitation care'.

This concerns both diagnostics and therapy carried out by supportive care disciplines. Rehabilitation care usually takes place after oncological treatment, but can also start during treatment (for example, preventive swallowing therapy). The standard oncological follow-up is not covered by this supportive care.

In this document you can indicate your answers digitally by checking the box that applies to you. Completing this survey takes 10 to 20 minutes. It is possible to save your answers in the meantime.

Thank you in advance for filling in!

[redacted]

• Are you a ☐ man ☐ woman

• What is your age? … ..Year

• Where do you work?

☐ Erasmus University Medical Center

- St. Elisabeth Hospital
- Leiden University Medical Center
- Haaglanden Medical Center
- Maastricht University Medical Center
- Netherlands Cancer Institute / Antoni van Leeuwenhoek hospital
- University Medical Center Groningen
- Leeuwarden Medical Center
- Radboud University Medical Center
- Rijnstate Hospital
- University Medical Center Utrecht
- Medisch Spectrum Twente
- VU University Amsterdam medical center
- Northwestern Hospital Group

• What is your profession?

- Head and neck surgeon
- Radiotherapist
- Oncologist
- Speech language pathologist
- Physiotherapist
- Dietitian
- Occupational therapist
- Art therapist
- Dentist
- Dental hygienist

- Prosthetics
- Psychiatrist
- Psychologist
- Medical social worker
- Rehabilitation Medical specialist
- Master Advanced Nursing Practitioner
- Oncology nurse
- Manager Planning & Control
- Employee Planning & Control department
- Other, namely ....

• How long have you been working in your department? ... . months / .... years

PART 1. REHABILITATION IN HEAD-NECK CANCER

• In this survey, "rehabilitation" is defined as: ‘care that focusses on functional, physical, psychological and social problems related to cancer, including supportive and rehabilitation care’. Given this definition; is there any provision of rehabilitation for head and neck cancer patients within your hospital?

□ Always □ Often □ Sometimes □ Rarely □ Never □ I don't know

• When does rehabilitation start? (Multiple answers possible)

□ Before the treatment

□ During the treatment

□ After the treatment

• Does provision of rehabilitation in your hospital depend on tumor group and stage?

□ Yes □ No □ I don't know □ Not Applicable

If so, to which patients do you provide rehabilitation? (Multiple answers possible)

Patients diagnosed with:

- T1 / T2 laryngeal cancer.
- T3 / T4 laryngeal cancer
- T1 / T2 oropharyngeal cancer
- T3 / T4 oropharyngeal cancer
- T1 / T2 oral cavity carcinoma
- T3 / T4 oral cavity carcinoma
- T1 / T2 nasopharyngeal cancer
- T3 / T4 nasopharyngeal cancer
- T1 / T2 paranasal sinus carcinoma.
- T3 / T4 paranasal sinus carcinoma
- T1 / T2 salivary gland tumors
- T3 / T4 salivary gland tumors
- Other, namely …………..

• Does provision of rehabilitation care in your hospital depend on HNC treatment?

□ Yes □ No □ I don't know □ Not Applicable

If so, which patients do you provide rehabilitation to? (Multiple answers possible)

Patients treated with:

- Surgery
- Radiotherapy
- Chemo radiation
- Photodynamic therapy
- Other, namely

Does your hospital have a guideline or protocol for the provision of rehabilitation by (supportive) care professionals?

- Yes, the national guideline on cancer rehabilitation
- Yes, the guideline of de Dutch Head and Neck Society
- Yes, a hospital wide protocol
- Yes, our own protocol in the department
- No.
- I don't know
- Other, namely ……………………………………………………………………

1a. SIGNALING AND REFERRAL

• Are functional problems or functional disorders in head and neck cancer patients identified in your hospital that require referral to (supportive) care providers?

□ Always □ Often □ Sometimes □ Rarely □ Never □ I don't know

Who performs this triage? (Multiple answers possible)

□ medical specialist □ nursing specialist □ nurse □ paramedic

□ Other, namely ....

If so, how is this triage assessed? (Multiple answers possible)

□ distress thermometer □ conversation □ distress thermometer + conversation □ I don't know

□ Other, namely ....

• Is there a moment of decision-making to determine whether mono- or multidisciplinary rehabilitation care is needed?

By multidisciplinary rehabilitation care we mean an integrated collaboration of the (supportive) care providers. If patients are treated separately by multiple (supportive) care providers, i.e. without mutual integrated cooperation, is seen as mono-disciplinary rehabilitation.

□ Always □ Often □ Sometimes □ Rarely □ Never □ I don't know

• Is a rehabilitation medical specialist involved in the rehabilitation process for patients with head and neck cancer?

□ Always □ Often □ Sometimes □ Rarely □ Never □ I don't know

If this is the case, what is his / her role in the rehabilitation process / rehabilitation treatment? (Multiple answers possible)

□ Indication of rehabilitation treatment

□ Intake rehabilitation treatment

□ Coordination rehabilitation treatment

□ For consultation concerning rehabilitation treatment

□ Other, namely ....

• Who refers the patient to (supportive) caregivers? (Multiple answers possible)

- Head and neck surgeon
- Radiotherapist
- Oncologist
- Speech language pathologist
- Physiotherapist
- Dietitian
- Occupational therapist
- Art therapist

- Dentist
- Dental hygienist
- Prosthetics
- Psychiatrist
- Psychologist
- Medical social worker
- Rehabilitation medical specialist
- Master Advance Nurse Practitioner
- Oncology nurse
- Other, namely ....
- No head and neck cancer patients are referred for rehabilitation

Explanation ....

To which (supportive) care providers do you refer to concerning rehabilitation for head and neck cancer patients? (Tick as appropriate) (Multiple answers possible)

	Within my own hospital	Primary care	To another hospital	I don't refer to rehabilitation	I don't know
Speech language pathologist					
Physiotherapist					
Orofacial therapist					
Lymphedema therapist					
Dietitian					
Occupational therapist					
Art therapist					
Dentist					
Dental hygienist					
Prosthetics					
Psychiatrist					
Psychologist					
Medical social worker					
Rehabilitation Physician					
MANP					
Oncology nurse					
Otherwise, namely……..					

Is there a rehabilitation department available in your hospital where rehabilitation/ supportive care for head and neck cancer patients can be provided?

□ Yes □ No □ I don't know

If yes, is the rehabilitation department in your hospital involved in the rehabilitation/ supportive care for psychosocial and / or physical problems of head and neck cancer patients?

- Yes, for patients' physical problems
- Yes, for patients' psychosocial problems
- Yes, for patients' physical and psychosocial problems
- No.
- I don't know

• Do you ever refer a patient to a rehabilitation center outside your hospital?

□ Always □ Often □ Sometimes □ Rarely □ Never □ I don't know

• How is referral to primary care organized in your hospital?

- Protocol-based, by the national guideline on cancer rehabilitation
- Protocol-based, by means of a hospital-wide protocol
- Protocol-based, we use our own protocol in the department
- Protocol- based, we use ………………………………………………… … ..
- Not protocol-based, I refer on indication
- I don't know
- Other, namely …………………………………… ……… ....

1b. INTAKE AND EVALUATION

• Do (supportive) caregivers set up and evaluate rehabilitation goals for patients with head and neck cancer?

- Always
- Often
- Sometimes
- Rarely
- Never
- I don't know
- If Yes, according to which method are these goals drawn up?

□ SMART (Specific, Measurable, Achievable, Relevant, Time-bound) □ ICF (International Classification of Functioning □ SAMPC (Somatic, Activities Daily Living, Social, Psychological, Communication model) □ I don't know □ Other, namely ………………………

Is there a standard multidisciplinary consultation within your hospital that takes place in a team regarding rehabilitation for head and neck cancer patients?

□ Always □ Often □ Sometimes □ Never □ I don't know

If this is the case - Which method is used (e.g. SAMPC / ICF)? ....

If so, which caregivers participate in this multidisciplinary rehabilitation consultation? (Multiple answers possible)

- Head and neck surgeon
- Radiotherapist
- Oncologist
- Speech language pathologist
- Physiotherapist
- Dietitian
- Occupational therapist
- Art therapist

- Dentist
- Dental hygienist
- Prosthetics
- Psychiatrist
- Psychologist
- Medical social worker
- Rehabilitation physician
- Master Advanced Nurse Practitioner
- Oncology nurse
- Other, namely ....
- No head and neck cancer patients are referred for rehabilitation

Explanation …………………………………………… …………………

If so, how often does this consultation take place? (Multiple answers possible)

- Monthly
- Weekly
- On indication
- Other, namely …………………… ……………

Explanation ……………………

Is rehabilitation care evaluated within your hospital (e.g. patient satisfaction)?

(Please comment)

□ Always □ Often □ Sometimes □ Never □ I don't know

If so, how is this care evaluated?

Explanation ....

....

• Which questionnaires are used in your hospital to assess quality of life?

- EORTC QLQ-Cancer30 (C30) and Head and Neck35 (H & N35)
- 36-Item Short Form Health Survey (SF-36)
- EuroQol-5dimensions (EQ-5D)
- I don't know
- Other, namely …………………………………………………………………………… …………

If so, for what purpose are these questionnaires administered?

- For scientific research
- For the purpose of Quality Registration (Dutch Head and Neck Audit)
- For effect evaluation
- For cost analysis
- The results are reported to the patient
- I don't know
- Other, namely ……………………………………………………………………… ………………

If so, at what times are the questionnaires administered? (Multiple answers possible)

- Baseline (diagnosis)
- 3 months after the end of treatment
- 6 months after the end of treatment
- 9 months after the end of treatment
- 12 months after the end of treatment
- 24 months after the end of treatment
- Other, namely ....

1c. INTERVENTIONS HEAD-NECK CANCER REHABILITATION

• Does your hospital have a guideline or protocol for the provision of rehabilitation by (supportive) care providers?

- Yes, the national guideline on cancer rehabilitation

- Yes, the Dutch HNC Allied Health Professionals working group (PWHHT)
- Yes, a hospital based protocol
- Yes, our own protocol in the department
- Yes, ……………………………………………………………… ……………………………
- No.
- I don't know
- Other, namely ………………………………………………………………………… …………

• For which intervention (s) are HNC patients referred to a speech language pathologist? (Multiple answers possible)

- Swallowing rehabilitation
- Voice rehabilitation
- Speech rehabilitation / articulation treatment
- Speech rehabilitation after laryngectomy
- Trismus treatment
- Olfactory rehabilitation after laryngectomy
- Hearing assessment
- Mime therapy
- Not applicable; patients are not referred to speech language pathologist
- I don't know
- Other,…………………………………………………………………………

- With what frequency are the above interventions performed on average by the speech language pathologist? (Multiple answers possible)

Intervention	On indication Patient	Frequency of treatment per week (e.g. 1/wk)	Duration of treatment in weeks (e.g. 12 wks)	Duration of session in minutes (e.g. 60 min)
Swallowing rehabilitation				
Voice rehabilitation				
Speech rehabilitation				
Speech rehabilitation after Laryngectomy				
Trismus therapy				
Olfactory rehabilitation after laryngectomy				
Hearing assessment				
Mime therapy				
Other, namely ……………………………				

• Which clinimetry is used by the speech language pathologist? (Tick as applicable)

	Purchased on indication	Standard assessment	No Assessment
Audiogram			
Tympanogram			
Swallowing video fluoroscopy			
Flexibel Endoscopic Evaluation of Swallowing (FEES)			
Maximal mouth opening (MMO)			
Functional Oral Intake Scale (FOIS)			
Swallowing Quality of Life (Swal-Qol)			
MD Anderson Dysfagia Inventory (MDADI)			
Eating Assessment Tool (EAT-10)			
Voice Handicap Index (VHI)			
Speech Handicap Index (SHI)			
Swallowing Outcomes After Laryngectomy (SOAL)			
Not applicable, patients are not referred to SLT			
I don’t know			
Other, namely, …………………...................................			

For which intervention (s) are HNC patients referred to a dietitian? (Multiple answers possible)

- ☐ Weight monitoring
- ☐ Monitoring full oral feeding
- ☐ Advice dietary supplements (drinking/ tube feeding)
- ☐ Nutritional advice during an exercise program
- ☐ General dietary advice
- ☐ Not applicable: patients are not referred to a dietitian

☐ I don't know

□ Other, namely ………………………………………………………… ..

……………………………………………………… ………………

- • With what frequency are the above interventions performed on average by the dietitian? (Multiple answers possible)

Intervention	On indication Patient	Frequency of treatment per week (e.g. 1/wk)	Duration of treatment in weeks (e.g. 12 wks)	Duration of session in minutes (e.g. 60 min)
Weight monitoring				
Monitoring sufficient nutrition				
Advice dietary supplements (drinking/ tube feeding)				
Nutritional advice during an exercise program				
General dietary advice				
Olfactory rehabilitation after laryngectomy				
Other, namely ……………………………				

• Which clinimetry is used by the dietitian? (Tick as applicable)

	Purchased on indication	Standard assessment	No Assessment
Muscle strength/ hand held dyno meter			
Short Nutritional Assessment Questionnaire (SNAQ)			
Body Mass index (BMI)			
Bio electrical impedance analysis (BIA)/ Bio electrical impedance spectroscopy (BIS)			
Not applicable, patients are not referred to dietitian			
I don’t know			
Other, namely, ………………….................................			

- For which intervention (s) are patients referred to physiotherapy? (Multiple answers possible)

☐ Improvement in fitness

☐ Muscle strength training

☐ Shoulder-neck exercise therapy

☐ Lymphedema therapy

- Trismus
- Not applicable: patients are not referred to physiotherapy
- I don't know
- Other, ………………………………………

• At what frequency are the above interventions used on average by physiotherapy? (Multiple answers possible)

Different……………………………

Intervention	On indication Patient	Frequency of treatment per week (e.g. 1/wk)	Duration of treatment in weeks (e.g. 12 wks)	Duration of session in minutes (e.g. 60 min)
Improving fitness/ exercise therapy				
Muscle strength training				
Trismus therapy				
Shoulder/ neck exercise therapy				
Lymphedema therapy				
Other, namely ………………………				

• Which clinimetry is administered by physiotherapy? (Tick as applicable)

	Purchased on indication	Standard assessment	No Assessment
Six minutes walking test (6MWT)			
Steep ramp test			
Shoulder Pain and Disability Index (SPADI)			
Active Range of Motion (AROM)			
Patient specific Complaints (PSK)			
Multidimensional Fatigue Index (MFI)			
Borg Rating of Perceived Exertion (Borg RPE scale)			
Maximum exercise test with ECG and breathing gas analysis			
Not applicable, patients are not referred to a physiotherapist			
I don’t know			
Other, namely, …………………...................................			

For which intervention (s) is referred to occupational therapy? (Multiple answers possible)

□ Sleep psycho-education

□ Psycho-education fatigue / energy coaching

□ Ergonomics

□ Return to work

□ Arm-hand function training

□ Cognitive rehabilitation

□ Training of daily activities

□ Not applicable: patients are not referred to occupational therapy

I don't know

□ Other, …………………………………

• With what frequency are the above interventions used on average by occupational therapy? (Multiple answers possible)

Intervention	On indication Patient	Frequency of treatment per week (e.g. 1/wk)	Duration of treatment in weeks (e.g. 12 wks)	Duration of session in minutes (e.g. 60 min)
Sleep psycho-education				
Psycho-education fatigue/ energy coaching				
Ergonomics				
Return to work				
Arm hand function training				
Cognitive rehabilitation				
Training of daily activities				
Not applicable: patients are not referred to occupational therapy				
I don’t know				
Other, namely ………………………				

• Which clinimetry is administered by occupational therapy? (Tick as applicable)

	Purchased on indication	Standard assessment	No Assessment
Canadian Occupational Performance Measure (COPM)			

Utrecht Scale for the Evaluation of Participation (USER-P)			
Impact on Participation and Autonomy (IPA)			
Patient specific Complaints (PSK)			
Multidimensional Fatigue Index (MFI)			
Not applicable, patients are not referred to an occupational therapist			
I don’t know			
Other, namely, …………………..................................			

- For which intervention (s) are patient referred to medical social work? (Multiple answers possible)

 - ☐ Psycho-education to cope with cancer
 - ☐ Return to work
 - ☐ Mindfulness
 - ☐ Psycho-education partner / loved ones
 - ☐ Cognitive behavioral therapy
 - ☐ Not applicable: patients are not referred to medical social work
 - ☐ I don't know
 - ☐ Other, …………………………………

• With what frequency are the above interventions used on average by the medical social worker? (Multiple answers possible)

Intervention	On indication Patient	Frequency of treatment per week (e.g. 1/wk)	Duration of treatment in weeks (e.g. 12 wks)	Duration of session in minutes (e.g. 60 min)
Psycho-education to cope with cancer				
Return to work				
Mindfulness				
Psycho-education partner/ loved ones				
Cognitive behavioral therapy				
Not applicable: patients are not referred to occupational therapy				
I don’t know				
Other, namely ………………………				

• Which clinimetry is used for medical social work?

(Tick as applicable)

	Purchased on indication	Standard assessment	No Assessment
Distress thermometer			
Hospital Anxiety Depression Scale (HADS)			
Center for Epidemiological Studies Depression Scale (CES-D)			
Not applicable, patients are not referred to an occupational therapist			
I don't know			
Other, namely, …………………..................................			

- For which intervention (s) are patient referred to Art therapy? (Multiple answers possible)

 Professional Art therapy

 Reactivation of daily activities

 Clarification of psycho-social supportive needs

 Not applicable: patients are not referred to art therapy

 I don't know

 Other,…………………… …………………………

• With what frequency are the above interventions used on average by the Art therapist? (Multiple answers possible)

Intervention	On indication Patient	Frequency of treatment per week (e.g. 1/wk)	Duration of treatment in weeks (e.g. 12 wks)	Duration of session in minutes (e.g. 60 min)
Professional Art therapy				
Reactivation of daily activities				
Clarification of psycho-social supportive needs				
Not applicable: patients are not referred to occupational therapy				
I don't know				
Other, namely………………………				

• Which clinimetry is used for Art therapy? (Tick as applicable)

	Purchased on indication	Standard assessment	No Assessment
Distress thermometer			
Hospital Anxiety Depression Scale (HADS)			
Center for Epidemiological Studies Depression Scale (CES-D)			
Not applicable, patients are not referred to an occupational therapist			
I don’t know			
Other, namely, …………………................................			

- For which intervention (s) are patients referred to a psychiatrist / psychologist? (Multiple answers possible)

  - ☐ Psychoeducation to deal with cancer
  - ☐ Psycho-education partner / loved ones
  - ☐ Psychological diagnostics
  - ☐ Psychic decompensation, medication
  - ☐ Cognitive behavioral therapy
  - ☐ Eye Movement Desensitization and Reprocessing (EMDR)
  - ☐ Not applicable: patients are not referred to psychiatry / psychology
  - ☐ I don't know
  - ☐ Other,……………………………………. ………………………

- With what frequency are the above interventions used on average by the psychiatrist/ psychologist?

  (Multiple answers possible)

Intervention	On indication Patient	Frequency of treatment per week (e.g. 1/wk)	Duration of treatment in weeks (e.g. 12 wks)	Duration of session in minutes (e.g. 60 min)
Psychoeducation to deal with cancer				
Psychoeducation partner/ loved ones				
Psychic decompensation, medication				
Cognitive behavioral therapy				
Psychological diagnostics				
Eye Movement Desensitization and Reprocessing (EMDR)				

Otherwise, namely ……………………………………… ….				

• Which clinimetry is taken by the psychiatrist / psychologist? (Tick as applicable)

	Purchased on indication	Standard assessment	No Assessment
Utrecht Coping List (UCL)			
Hospital Anxiety Depression Scale (HADS)			
Center for Epidemiological Studies Depression Scale (CES-D)			
Symptom Checklist (SCL-90)			
Not applicable, patients are not referred to an occupational therapist			
I don’t know			
Otherwise, namely, ………………….................................			

PART 2. FINANCIAL ASPECTS

1. REIMBURSEMENT OF REHABILITATION

• Does your hospital use a separate reimbursement in Dutch DBC (Diagnose Behandel Combinatie = Diagnosis Treatment Combination) for rehabilitation? (Please comment)

- Yes
- No
- I don't know
- Other, ...

Explanation

……… ………… ………………………………. ………………………………………………………………………………

• Can you indicate how the costs for the (supportive) care disciplines in the context of rehabilitation for patients with head and neck cancer are declared in your hospital? (Tick as applicable)

- Dutch rehabilitation group imbursement (DRG) in Dutch Diagnose Behandel Combinatie (DBC)

- Primary care costs
- Project subsidies
- Other I don't know

• Does the financing of care activities related to rehabilitation for patients with head and neck cancer in your hospital cover? (Please comment)

- Yes
- No
- I don't know

Explanation

……

………………………………………………………………………………… ……………………………….

- Are there restrictions in your hospital regarding the provision of rehabilitation for patients with head and neck cancer, which arise from financial considerations? (Please comment)

- Yes
- No
- I don't know

Explanation

……

………… ………………………………. ……………………………………………………………………

• How do you think rehabilitation care for patients with head and neck cancer that you would like to provide additionally should be reimbursed? (Please comment)

- By health insurance
- By the government

- By a fund / foundation
- From a personal contribution
- By a project subsidy
- Other, namely …………… ..

Explanation

………

………… ………………………………. …………………………………………………………………………

• Is it sometimes the case that rehabilitation care you provide is not reimbursed, as a result of which the patient has to pay per consultation or is referred to primary care? (Please comment)

- Yes, patients have to pay per consultation
- Yes, patients are referred to primary care
- No.
- I don't know

Explanation ……………………………………

PART 3. FACTORS AFFECTING THE PROVISION OF REHABILITATION

Below you will find a number of factors that can influence the provision of rehabilitation for head and neck cancer patients.

• Can you indicate to what extent you experience the following factors in your situation as facilitator or barrier? If there are any aspects that you consider important in this recital, you can complete them in the list. When we talk about the "(supportive) care providers", this concerns the (supportive) care providers in your hospital.

Clinical Factors (filled in by all health care professionals)

FACTORS	Grol/ Wensing	CTA	Barrier	Facilitator	Not applicable	Remark
(Interim) evaluation of the effect of interventions to tailor the rehabilitation to the outcomes of the patient		Outcomes/effect on population				

Providing evidence-based rehabilitation (e.g. according to a guideline)	Cognitive	Efficacy/effectiveness				
Evaluation of evidence-based rehabilitation		Efficacy				
Expertise / knowledge of specialists and (supporting) care providers	Education					
Attitude of the specialists and (supportive) care providers	Attitude					
Motivation of the specialists and (supportive) care providers	Motivation					
Access to information about rehabilitation / insight for the referrer	Education	dissemination				

ECONOMICAL FACTORS (filled in by Medical specialist)

FACTORS	Grol/Wensing	CTA	Barrier	Facilitator	Not applicable	Remark
Degree of reimbursement of rehabilitation (e.g. all necessary rehabilitation is included in DBC is very conducive to providing rehabilitation)	Reimbursement					
Cost-effectiveness of rehabilitation interventions		Cost-effectiveness				

ECONOMICAL FACTORS (filled in by managers planning & control)

FACTOREN	Grol/Wensing	CTA	Barrier	Facilitator	Not applicable	Remark
Rate for rehabilitation negotiated with the health insurer (e.g. the rate is covering or inadequate)						
General subsidy / financial support within your hospital to provide care						

Contractual agreements between health insurers and institutions						

PATIENT-RELATED FACTORS (filled in by all health care professionals)

FACTOREN	Grol/Wensing	CTA	Barrier	Facilitator	Not applicable	Remark
Intellectual level of patients' health skills	Cognition					
Availability of rehabilitation information for the patient	Education					
Perception of patient acceptance?	Attitude	Acceptibility				
Confidence in specialists and (supportive) care providers	Attitude					
Motivation / needs of the patient with regard to rehabilitation	Motivation					
Prioritizing the Motivation rehabilitation process	Motivation					
Patient expectations regarding recovery and rehabilitation		Psychological reactions				
Travel distance		Social and environmental				
Availability of transportation to and from the hospital		Social and environmental				
Financial charges (e.g. travel expenses, absence from work)		Social and environmental				
Proficiency in the Dutch language (e.g. language barrier)		Social and environmental				
Time schedule rehabilitation in relation to other work-related and social activities of the patient						
Self-management ◊ health skills						
Nature of the disease or treatment (e.g. curative or palliative disease)						

Social support		Social and environmental				
Psychiatric history / co-morbidity		Psychological reactions				
Casemanager contact person		Patient-centeredness				

ORGANISATIONAL FACTORS (filled in by all health care professionals)

FACTOREN	Grol/Wensing	CTA	Barrier	Facilitator	Not applicable	Remark
National guideline for cancer rehabilitation	Arrangements	Organizational implementation				
Availability of a protocol in your hospital	Arrangements	Organizational implementation				
Availability of specialists and (supportive) care providers	Capacity	Accessibility				
Coordinating the interventions between the (supportive) care providers (logistics)						
Timely inventory according to needs of rehabilitation and provision of rehabilitation		Accessibility				
Screening of patients before rehabilitation is provided		Skills/routines				
Time management with regard to the provision of rehabilitation						
Communication between the specialists and (supportive) care providers						
Availability of spaces for providing rehabilitation	Capacity					
Spatial distances between specialist and (supportive) care providers (e.g. integrated practice units)	Capacity	Accessibility				
Availability of facilities to provide	Capacity	Accessibility				

rehabilitation (e.g. measuring instruments)						
Collaboration with primary care						
Specialization of (supportive) caregivers regarding head and neck rehabilitation		Skills/routines?				
Availability of rehabilitation training for specialists and (supporting) care providers		Educational/training				

- What is your opinion about offering a multidisciplinary rehabilitation program for patients with head and neck cancer? (Tick as applicable)

  - ☐ A multidisciplinary rehabilitation program seems to me an asset for patients with head and neck cancer.
  - ☐ I am satisfied as the rehabilitation is organized within our hospital and see no added value in a multidisciplinary rehabilitation program for patients with head and neck cancer.
  - ☐ It is not possible to offer a multidisciplinary rehabilitation program in my hospital, because …………………………………………………… ………………………….
  - ☐ We already apply a multidisciplinary rehabilitation program for patients with head and neck cancer.
  - ☐ I don't know
  - ☐ Other, namely ... .

• Are you satisfied with the way in which rehabilitation for patients with head and neck cancer is organized in your hospital?

  - ☐ Very satisfied
  - ☐ Satisfied
  - ☐ Not satisfied / not dissatisfied
  - ☐ Dissatisfied
  - ☐ Very dissatisfied

• What do you think could be improved about the rehabilitation in your hospital? (Please comment)

Explanation ……

……

……
