# Supplementary material for: Organization of head and neck cancer rehabilitation care: a national survey among healthcare professionals in Dutch head and neck cancer centers
Source: Eur Arch Otorhinolaryngol. 2024 Feb 7;281(5):2575–85. doi: 10.1007/s00405-024-08488-1 (PMC11023954; doi:10.1007/s00405-024-08488-1)
Supplement: Supplementary file 2 — Supplementary file2 (PDF 418 KB) [file 405_2024_8488_MOESM2_ESM.pdf]

Appendix B - An overview of the categories and items included in the survey to assess the barriers and facilitators of head and neck cancer rehabilitation care provision

No.	Category	Item
1	Clinician-related	Attitude of allied health professionals towards head and neck cancer rehabilitation
2		Attitude of medical specialists towards head and neck cancer rehabilitation
3		Effect evaluation of head and neck cancer rehabilitation
4		Evidence-based head and neck cancer rehabilitation interventions
5		Expertise/knowledge of medical specialists and allied health professionals
6		Knowledge of health professionals (in general) on head and neck cancer rehabilitation
7		Knowledge of referrer on (in- and external) head and neck cancer rehabilitation referral options
8		Knowledge of referrer on head and neck cancer rehabilitation
9		Motivation of allied health professionals to provide head and neck cancer rehabilitation
10		Motivation of medical specialists to provide head and neck cancer rehabilitation
11		Stimulation of patient participation head and neck cancer rehabilitation by health professionals
1	Economic: Part 1	Coverage structure for head and neck cancer rehabilitation
2		Evaluation of cost-effectiveness of head and neck cancer rehabilitation
3	Economic: Part 2*	Contractual agreements between health insurers and hospital
4		Financial support or subsidy within the hospital
5		Tariffs negotiated with the health insurers
1	Patient-related	Availability of patient information on head and neck cancer rehabilitation
2		Expectations of patients regarding head and neck cancer rehabilitation
3		Financial capacity of patients
4		Health literacy of patients
5		Language proficiency of patients
6		Motivation and therapy compliance of patients
7		Prioritization of head and neck cancer rehabilitation care by patients
8		Psychiatric history and/or comorbidity
9		Social safety net/informal care for patients
10		Stimulation of patients by relatives and friends
11		Time for head and neck cancer rehabilitation in relation to social- and work-related activities
12		Transport to/from the hospital
13		Travel time to the hospital
1	Organizational	Accessibility of materials to provide head and neck cancer rehabilitation (e.g. instruments)
2		Alignment of interventions of allied health professionals
3		Applying the national cancer rehabilitation guideline
4		Available locations to provide head and neck cancer rehabilitation
5		Availability of a contact person
6		Capacity of staff including medical specialists and allied health professionals
7		Collaboration with health care professionals in primary care
8		Communication between medical specialists and allied health professionals
9		Educational opportunities for health professionals regarding head and neck cancer rehabilitation
10		Physical distance between medical specialists and allied health professionals (e.g. lack of integrated practice units)
11		Planning of head and neck cancer rehabilitation
12		Protocol to provide head and neck cancer rehabilitation
13		Specialized allied health professionals in head and neck cancer rehabilitation
14		Timely inventory of head and neck cancer rehabilitation needs (screening)

Healthcare professionals reflect the persons who provide supportive care.
*Completed by the managers/employees of the Financial Department.
