# Supplementary material for: Organization of head and neck cancer rehabilitation care: a national survey among healthcare professionals in Dutch head and neck cancer centers
Source: Eur Arch Otorhinolaryngol. 2024 Feb 7;281(5):2575–85. doi: 10.1007/s00405-024-08488-1 (PMC11023954; doi:10.1007/s00405-024-08488-1)
Supplement: Supplementary file 3 — Supplementary file3 (PDF 211 KB) [file 405_2024_8488_MOESM3_ESM.pdf]

Appendix C – protocols and/or guidelines used in rehabilitation care provision in Dutch head and neck centers

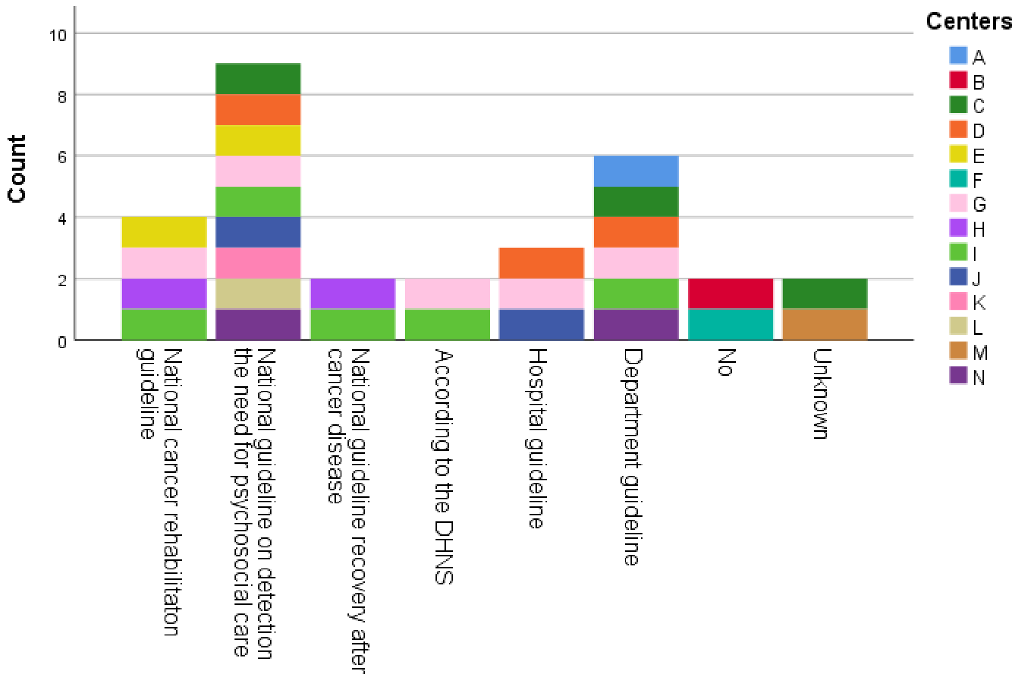

Abbreviations: DHNS – Dutch head and neck society
