# Supplementary material for: Organization of head and neck cancer rehabilitation care: a national survey among healthcare professionals in Dutch head and neck cancer centers
Source: Eur Arch Otorhinolaryngol. 2024 Feb 7;281(5):2575–85. doi: 10.1007/s00405-024-08488-1 (PMC11023954; doi:10.1007/s00405-024-08488-1)
Supplement: Supplementary file 4 — Supplementary file4 (PDF 653 KB) [file 405_2024_8488_MOESM4_ESM.pdf]

Appendix D - Overview of allied health care in 14 Dutch HNC centers: Availability of AHPs, rehabilitation interventions and clinimetrics

**Availability healthcare professional**	**Clinimetrics and interventions**	**Centers**													
**Allied health professional (% of centers)**		**A**	**B**	**C**	**D**	**E**	**F**	**G**	**H**	**I**	**J**	**K**	**L**	**M**	**N**
**Speech language therapist (100)**		X	X	X	X	X	X	X	X	X	X	X	X	X	X
Interventions	Swallowing rehabilitation	A	A	A	A	R	A	A	R	A	A	A	R	R	A
	Voice rehabilitation	A	R	R	A	R	A	A	R	A	A	R	R	R	R
	Speech and articulation rehabilitation	A	R	A	A	R	A	A	R	A	R	R	R	R	R
	Speech rehabilitation after TLE	A	A	A	A		A	A	R	A	A	A	A	A	R
	Trismus therapy		A	R	A	R	A	A					R	R	
	Olfactory rehabilitation after TLE	A	A	R	A		A	A		R	R	A	R	A	R
	Mime therapy	R		R	R	R		A		R	R		R	R	R
Clinimetrics	Swallowing video / FEES	I	I	I	I	I	I	A	I	I	I	A	I	I	I
	MMO			I	I	I		A		A		A	I	I	
	FOIS	A	I	A	A	A	A	A	I	A	I		I	A	A
	Swal-QoL		I	I	I		I	A			I			A	A
	MDADI		I		I		I	A							
	EAT-10		I			I	I		A						
	VHI	I	A	I	I	A		A	I	I	I		I	A	A
	SHI				I	I		A		I	I		I	I	
	SOAL							A							
**Allied health professional (% of centers)**		**A**	**B**	**C**	**D**	**E**	**F**	**G**	**H**	**I**	**J**	**K**	**L**	**M**	**N**
**Dietitian (93)**		X	X		X	X	X	X	X	X	X	X	X	X	X
Interventions	Monitoring weight	A	A		A	A	A	A	A	A	A	A	A	R	A
	Monitoring intake	A	A		P	A	A	A	A	A	A	A	A	R	A
	Advice on nutritional supplements	A	R		A	A	A	A	A	A	A	A	A	R	A
	Nutritional advice in general	A	A		P	A	A	a	A	A	A	A	A	R	A
	Nutritional advice during a physical program						R	R							

Clinimetrics	Pinch strength test	I	A				I	I	A	A	I			A	
	SNAQ					A	A	A			I				A
	BMI	A	A			A	A	A	A	A	A	A	A	A	A
	BIS		I				I	I	A	I				A	
	PG-SGA							I							
**Allied health professional (% of centers)**		**A**	**B**	**C**	**D**	**E**	**F**	**G**	**H**	**I**	**J**	**K**	**L**	**M**	**N**
**Physiotherapist (86)**		X	X		X	X	X	X		X	X	X	X	X	X
Interventions	Improving physical condition	A	R		A			A			A	R	R	R	R
	Muscle strength training	A	R		A			A			A	R	R	R	A
	Trismus therapy				R					A		R		R	A
	Shoulder and neck exercise training	A	R		A			A		A	A	A	A	A	A
	Lymphedema therapy	R			R			A		A		R	R		R
Clinimetrics	6MWT				I			I		A	I		I	I	
	Steep ram test				I			A			I		A	I	
	SPADI				I			I		A	A	I			I
	AROM	A	A		I			I		A	A	A	A	A	A
	MFI				I			A		I	I				
	PSC				I			A			I	I	A		I
	Borg RPE-scale	I			I			A			A		A		A
	Maximal exercise test with ECG and breath gas analysis				I			I							
**Allied health professional (% of centers)**		**A**	**B**	**C**	**D**	**E**	**F**	**G**	**H**	**I**	**J**	**K**	**L**	**M**	**N**
**Medical social worker (57)**		X			X		X	X			X	X	X	X	
Interventions	PE coping with cancer disease	R			R		R	R			R	A	A	R	
	PE partner/loves ones	R			R		R	R			R	A	A	R	
	Resumption of work	R			R		R	R			R	R	A	R	
	Mindfulness							R					A	R	
	Cognitive behavioral therapy				R			R			R		A	R	
Clinimetrics	Distress thermometer						A	A			A			I	
	HADS						I	A						I	
	CED-D						I								

**Allied health professional (% of centers)**		**A**	**B**	**C**	**D**	**E**	**F**	**G**	**H**	**I**	**J**	**K**	**L**	**M**	**N**
**Psychologist (50)**		**X**		**X**	**X**	**X**	**X**	**X**				**X**			
Interventions	PE coping with cancer disease	R		R	R	R	A	R				R			
	PE partner/loved ones			R	R	R	P	R				R			
	Psychological decompensation	R		R	R	R	R					R			
	Cognitive behavioral therapy	R		R	R	R	R	R				R			
	Psychological diagnostics	R		R	R	R	R	R				R			
	EMDR	R		R	R	R	R	R				R			
Clinimetrics	UCL	I		I		I		A				I			
	HADS	I		I			I	A				I			
	CES-D			I		I	I					I			
	SCL-90	I		I		I		A							
**Allied health professional (% of centers)**		**A**	**B**	**C**	**D**	**E**	**F**	**G**	**H**	**I**	**J**	**K**	**L**	**M**	**N**
**Psychiatrist or psychiatric nurse (specialist) (36)**		**X**		**X**	**X**			**X**		**X**					
Interventions	PE coping with cancer disease	R		R				R							
	PE partner/loved ones	R		R	R			R		R					
	Psychological decompensation/medication	A		A	A			A		R					
	Cognitive behavioral therapy									R					
	Psychiatric diagnostics	R		A	A			R		R					
	Drug rehabilitation	R		A	A			R							
Clinimetrics	UCL														
	HADS														
	CED-D														
	SCL-90			I											
**Allied health professional (% of centers)**		**A**	**B**	**C**	**D**	**E**	**F**	**G**	**H**	**I**	**J**	**K**	**L**	**M**	**N**
**Occupational therapist (21)**								**X**		**X**		**X**			
Interventions	PE on sleep							A		R					
	PE on fatigue/energy coaching							A		R					
	Ergonomics							A		R		R			

	Resumption of work							A		R					
	Arm-hand function training							A				R			
	Cognitive rehabilitation							A				R			
	Training of ADL							A		R		R			
Clinimetrics	COPM							A		I					
	USER-P									I					
	IPA														
	PSC							A							
	MFI							A							
**Allied health professional (% of centers)**		**A**	**B**	**C**	**D**	**E**	**F**	**G**	**H**	**I**	**J**	**K**	**L**	**M**	**N**
**Art therapist (14)**		x						x							
Interventions	Art therapy	R						R							
	Reactivating daily activity	R													
	Inventory patient's medical queries	R						A							
	Other														
Clinimetrics	HADS							A							
	CES-D														

Legends:

x means the healthcare professional is available in HNC rehabilitation; empty cells indicate unavailability.

A means interventions and clinimetrics are applied either as standard care or according to protocol or guideline.

I means interventions and clinimetrics are applied indicated by needs assessment.

R means interventions and clinimetrics are applied by referral.

An empty or white cell reflects not known or not applicable.

Abbreviations: 6 MWT, 6 minutes walking test; ADL, activities of daily living; AROM, active range of motion; Borg RPE-scale, borg rating of perceived exertion; BMI, body mass index; BIS, bio-electric impedance spectroscopy; CES-D, center or epidemiological studies depression scale; COPM, Canadian Occupational Performance Measure; EAT-10, Eating Assessment Tool; ECG, electrocardiogram; FEES, flexible endoscopic evaluation of swallowing; FOIS, functional oral intake scale; HADS, hospital anxiety depression scale; IPA, impact of participation and autonomy; MDADI, MD Anderson dysphagia inventory; MMO, maximal mouth opening; MFI, multidimensional fatigue index; PE, psycho-education; PG-SGA, patient-generated subjective global assessment; PSC, patient specific complaints; SCL-90, symptom checklist-90; SHI, speech handicap index; SLP, speech-language pathologist; SNAQ, short nutritional assessment questionnaire; SOAL, swallowing outcomes after laryngectomy; Swal-Qol, swallowing quality of life; SPASI, shoulder pain and disability index; TLE, total laryngectomy; UCL, Utrecht coping list; USER-P, Utrecht scale for evaluation of rehabilitation-participation; VHI, voice handicap index.
